# Galacto-Oligosaccharides Exert Bifidogenic Effects at Capsule-Compatible Ultra-Low Doses

**DOI:** 10.3390/metabo15080530

**Published:** 2025-08-05

**Authors:** Lucien F. Harthoorn, Jasmine Heyse, Aurélien Baudot, Ingmar A. J. van Hengel, Pieter Van den Abbeele

**Affiliations:** 1 Clasado Biosciences Ltd., Imperium Building, Imperial Way, Worton Grange, Reading RG2 0TD, UK; lucien.harthoorn@clasado.com; 2 Cryptobiotix SA, Technologiepark-Zwijnaarde 82, 9052 Ghent, Belgium; jasmine.heyse@cryptobiotix.com (J.H.); aurelien.baudot@cryptobiotix.com (A.B.); ingmar.vanhengel@cryptobiotix.com (I.A.J.v.H.)

**Keywords:** prebiotic, GOS, SCFA, *Bifidobacteriaceae*, *Lachnospiraceae*, SIFR^®^ technology

## Abstract

Background: Prebiotics are selectively used by host microorganisms to promote health. Because effective prebiotic doses (1.5–30 g/day) often require inconvenient delivery formats, this study aims to explore whether capsule-compatible doses of galacto-oligosaccharides (GOS) can effectively modulate the gut microbiome. Methods: The impact of Bimuno^®^ GOS (Reading, UK) at 0.5, 0.75, 1.83, and 3.65 g on the adult gut microbiome was assessed using the ex vivo SIFR^®^ technology (*n* = 8), a clinically validated, bioreactor-based technology. Results: The GOS were rapidly fermented and significantly increased beneficial *Bifidobacterium* species (*B. adolescentis*, *B. bifidum,* and *B. longum*), even at the lowest tested dose. In doing so, GOS strongly promoted SCFA production, particularly acetate (significant from 0.5 g) and butyrate (significant from 0.75 g). Gas production only mildly increased, likely as *Bifidobacterium* species do not produce gases. Based on the ability of the SIFR^®^ technology to cultivate strictly anaerobic, hard-to-culture gut microbes, unlike in past in vitro studies, we elucidated that GOS also enriched specific *Lachnospiraceae* species. Besides *Anaerobutyricum hallii*, this included *Bariatricus comes*, *Blautia* species (*B. massiliensis*, *Blautia_A*, *B. faecis*), *Oliverpabstia intestinalis*, *Mediterraneibacter faecis,* and *Fusicatenibacter* species. Finally, GOS also promoted propionate (significant from 0.75 g), linked to increases in *Phocaeicola vulgatus*. Conclusions: GOS displayed prebiotic potential at capsule-compatible doses, offering greater flexibility in nutritional product formulation and consumer convenience. Notably, the strong response at the lowest dose suggests effective microbiome modulation at lower levels than previously expected.

## 1. Introduction

The gastrointestinal tract hosts a complex microbiome, whose composition and metabolic activity shape host physiology. Through the fermentation of dietary components, the gut microbiome produces short-chain fatty acids (SCFAs) and other metabolites that influence the immune and nervous systems [1,2,3]. As dysbiosis (= disruption of the microbiome) has been linked to non-communicable diseases such as obesity, diabetes, allergies, inflammatory bowel disease, and neuropsychiatric conditions [4,5,6], maintaining microbial homeostasis is essential for overall health. One approach to modulate the microbiome is the use of prebiotics. Prebiotics are dietary substrates selectively utilized by host microorganisms to confer performance or health benefits [7,8]. They are minimally digested in the upper gastrointestinal tract [9,10] and fermented by colonic microbes, enhancing their growth and metabolic activity. Common prebiotics include fructans, galacto-, xylo-, and chito-oligosaccharides, as well as lactulose, resistant starch, and polyphenols [11]. Galacto-oligosaccharides (GOS) are widely used and are composed of galactose oligomers [12]. GOS are selectively fermented by gut bacteria, particularly members of the genus *Bifidobacterium* [13,14]. In addition to their bifidogenic effects, GOS can modulate the overall microbiota composition and activity [14,15,16], enhance gut comfort [15,17], modulate immune function [18,19], improve nutrient absorption (e.g., calcium [20] and iron [21]), reduce stress-related symptoms [22] and neuroinflammation [23], and support gut barrier integrity [24]. Effective prebiotic doses span a large range from 1.5 up to 30 g per day [8]. However, at high doses, prebiotics can induce temporary gastrointestinal discomfort (e.g., abdominal pain, bloating, and gas) in both healthy individuals and those with gastrointestinal disorders such as irritable bowel syndrome (IBS) or Crohn’s disease [25,26,27,28,29,30]. In addition to tolerability concerns, many doses pose practical limitations, as capsules or tablets can only hold smaller volumes, necessitating administration via sachets instead, for instance.

Bimuno^®^ GOS (also referred to as B-GOS) is a well-characterized GOS mixture which has been shown to exert beneficial effects in several clinical trials at doses of 1.37–7 g/d [14,17,18,31,32]. While these studies have provided valuable evidence supporting the efficacy of Bimuno^®^ GOS, these trials inherently involved dietary and lifestyle confounders that complicate mechanistic interpretation. Two studies with in vitro fermentation models have shown that Bimuno^®^ GOS enriches *Bifidobacteriaceae* and certain genera of *Bacteroidaceae* [33,34]. However, these in vitro simulations particularly captured changes in Actinomycetota (e.g., *Bifidobacteriaceae*) and Bacteroidota (e.g., *Bacteroidaceae*), while they tended to under-represent many Bacillota (e.g., *Lachnospiraceae* and *Ruminococcaceae*), which may limit their ability to fully reflect community shifts that occur in vivo. Additionally, the response to prebiotic treatments can vary substantially between individuals. For example, Holmes et al. (2022) showed that individual identity, rather than prebiotic type, was the major determinant of SCFA response to prebiotic interventions [35]. Similarly, different studies described responders and non-responders to GOS treatments [13,36,37]. This stressed the importance of addressing interpersonal differences in preclinical research, an aspect that is often overlooked.

In this study, we therefore employed the ex vivo SIFR^®^ technology to evaluate the impact of Bimuno^®^ GOS at capsule-compatible doses (0.5, 0.75, 1.83, and 3.65 g) on the gut microbiota of eight adults. The SIFR^®^ technology is validated to generate insights that correspond to clinical trial outcomes observed after repeated product intake over several weeks, including shifts in microbiome composition [38] and changes in gut barrier function and immunity [39]. This originates from the ability of the technology to reliably cultivate the full diversity of gut microbes [38]. Moreover, the high throughput enables the inclusion of a larger number of donors compared to conventional gut models, thereby allowing assessment of inter-individual variability in treatment responses. The key objectives of the study were to improve understanding of prebiotic effects of Bimuno^®^ GOS and to quantify the minimal effective (capsule-compatible) dose.

## 2. Materials and Methods

### 2.1. Selection Criteria Test Subjects

To decipher the impact of different doses of GOS treatments on ex vivo cultivated microbiomes, fresh faecal samples were collected, adhering to the following selection criteria: age between 25 and 65 years, no antibiotic treatment in the past 3 months, non-smoking status, alcohol consumption of <3 units/day, no gastrointestinal disorders (cancer, ulcers, inflammatory bowel disease), and a body mass index of 20 to 25. The consistency of faecal samples was assessed via the Bristol stool scale [40], which confirmed that all samples were within the normal range (BSS = 3–4; average = 3.9). Four male and four female subjects were enrolled with an average age of 37.4 (±8.6) years: 1 (f, 48.2), 2 (m, 47.8), 3 (f, 30.3), 4 (m, 28.3), 5 (f, 26.7), 6 (f, 42.7), 7 (m, 40.9), and 8 (m, 34.2). Each participant signed an informed consent form to agree to the anonymous use of their sample for the present study, which was approved by the Ethical Committee of the University Hospital Ghent (reference number BC-09977).

### 2.2. Ex Vivo Intestinal Fermentation Assay (SIFR^®^): Study Configuration, Timeline, and Analysis

The SIFR^®^ technology is a bioreactor-based, faecal fermentation technology designed to closely mimic the human intestinal environment while maintaining the original donor’s faecal microbiome. Unlike traditional in vitro systems that rapidly lose microbial complexity, the SIFR^®^ technology preserves hard-to-culture species, thereby offering more physiologically relevant insights into microbiome responses to interventions. Ex vivo SIFR^®^ experiments were executed in accordance with those carried out previously by Van den Abbeele et al. (2023) [38]. Briefly, bioreactors were filled with 5 mL of a blend of nutritional medium (M0017, Cryptobiotix, Ghent, Belgium), the faecal inoculum from an individual subject, and a specific GOS dose. Reactors were sealed and made anaerobic using a bioreactor management device (Cryptobiotix, Ghent, Belgium). The bioreactors were incubated under constant agitation (140 rpm) at 37 °C (MaxQ 6000, Thermo Scientific, Merelbeke, Belgium).

**Figure 1:**
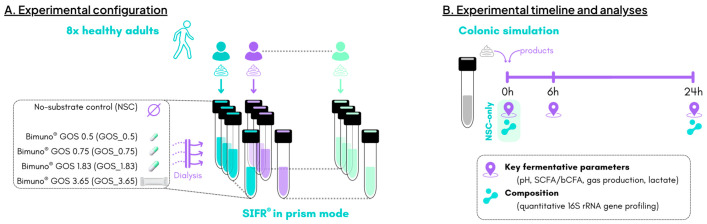
**The impact of four doses of the GOS preparation was assessed on the human adult gut microbiota using the ex vivo SIFR^®^ technology (n = 8).** (**A**) Experimental configuration; (**B**) timeline and analysis.

Five study arms were tested for each human adult (n = 8), consisting of an unsupplemented parallel control (no substrate control; NSC) and four doses of Bimuno^®^ GOS powder (equivalent of a daily intake of 0.5, 0.75, 1.83, and 3.65 g), which was provided by Clasado Biosciences Ltd. (Reading, UK) (Figure 1A). Bimuno^®^ GOS mainly consists of β-1,3-linkages and is a mixture of oligosaccharides with a degree of polymerization (DP) of 2 to 9 with DP3 as the major fraction (>40%). Comparing the outcomes of GOS-treated study arms with the NSC condition (within a given time point: 6 or 24 h) allowed for the establishment of treatment effects as the NSC is a condition in which the faecal microbiomes are grown under identical conditions (e.g., background medium and time), except for GOS supplementation. At 0 h (baseline), 6 h, and 24 h, gas pressure was measured in the headspace of the reactors and liquid samples were collected for the assessment of key fermentative parameters (pH, gas, SCFA, and branched-chain fatty acid (bCFA) production) and microbial composition (Figure 1B). The NSC study arm was run in triplicate for each individual subject to confirm the high technical reproducibility.

### 2.3. Key Fermentation Parameters

SCFAs (acetate, propionate, and butyrate) and bCFAs (sum of isobutyrate, isocaproate, and isovalerate) were quantified upon diethyl ether extraction using a gas chromatograph with flame ionization detection (Trace 1300 chromatograph; Thermo Fisher Scientific, Merelbeke, Belgium), as previously described [41]. Briefly, liquid samples were diluted in distilled water (1:3) and acidified (using sulfuric acid), after which sodium chloride, 2-methylhexanoic acid (internal standard), and diethyl ether were added. The pH was determined using an electrode (Hannah Instruments Edge HI2002, Temse, Belgium) and lactate was measured via an enzymatic method (Enzytec^TM^, R-Biopharm, Darmstadt, Germany).

### 2.4. Taxonomic Microbiota Analysis by Quantitative 16S rRNA Gene Profiling

Quantitative insights into microbial composition were obtained by combining two methods, i.e., relative abundances (%; 16S rRNA gene sequencing) were multiplied with total cell numbers (cells/mL; flow cytometry) to estimate the cells densities (cells/mL) in each individual sample for different taxonomic levels (phylum, family, genus, and OTU (operation taxonomic unit) level).

To determine relative abundances, DNA was first extracted via the SPINeasy DNA Kit for Soil (MP Biomedicals, Eschwege, Germany), after which library preparation and sequencing were performed on an Illumina MiSeq platform with v3 chemistry (2 × 300 bp). Amplicons (of around 425 bp) were generated via the primers 341F (50-CCT ACG GGN GGC WGC AG-30) and 785Rmod (50-GAC TAC HVG GGT ATC TAA KCC-30), which target the V3–V4 region of the 16S rRNA gene. Pre-processing and OTU picking was performed with Mothur v1.35.1 [42] (including removal of sequences containing ambiguous bases, with homopolymer stretches of more than 8 bases or with a Phred quality score below 33, alignment against the 16S Mothur-Silva SEED r138 reference alignment, with elimination of chimera with the uchime algorithm, OTU picking by clustering at the 97% identity level, removal of singleton sequences to reduce spurious OTU formation, and creation of OTU count tables (in the BIOM format); annotation of representative sequences was carried out with NCBI blast v2.10.0 [43]. To quantify total cell numbers, samples were diluted in anaerobic PBS, after which microbial cells were stained with SYTO 16 and counted via a Novocyte flow cytometer (Agilent, Santa Clara, CA, USA).

### 2.5. Data Analysis

All analyses were performed using R (version 4.4.0; www.r-project.org; accessed on 25 April 2025). First, a series of violin plots, bar charts, and heat maps were constructed (ggplot2 package 3.5.1). While violin plots and bar charts present the actual values, heat maps show log_2_-transformed fold changes for treatments compared to the reference (NSC). Hence, values of >0 reflect a stimulation on GOS treatment, while values of <0 indicate an inhibition by GOS treatment. Statistical analysis used a linear mixed-effects model, with treatment as a fixed effect and donor identity included as a random effects (lmerTest package v3.1-3). Adjustments for multiple comparisons were performed using the Benjamini–Hochberg correction (stats package 3.6.2). Treatment effects of specific GOS doses compared to the reference (NSC) were assessed via a post hoc pairwise comparison, again with Benjamini–Hochberg correction of *p*-values. Effects were considered to be significant at *p*_adjusted_ < 0.05. In the violin plots (key fermentative parameters) and stacked bar charts (community composition), statistical differences between specific doses of GOS powder and the NSC are indicated with * (0.01 < *p*_adjusted_ < 0.05), ** (0.001 < *p*_adjusted_ < 0.01), or *** (*p*_adjusted_ < 0.001). In the violin plots, the ranks of average values per study arm are indicated below the statistical indicator, with the lowest and highest values being highlighted in purple and yellow, respectively. Finally, pairwise correlation analysis using Spearman’s rank correlation coefficient was performed between SCFA production and absolute levels of different families.

## 3. Results

### 3.1. Differences in Microbiome Composition at Baseline

**Figure 2:**
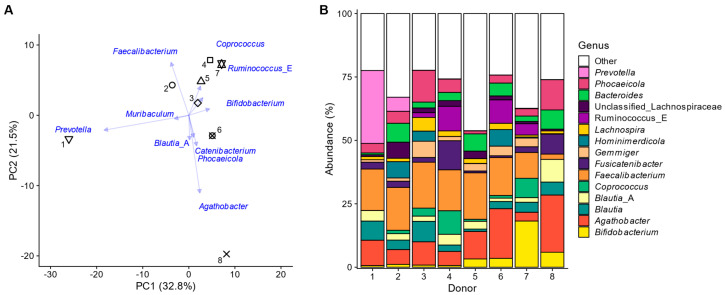
**The faecal microbiota of the eight human adults displayed interpersonal differences.** (**A**) Principal component analysis based on centred abundances at genus level (%) with indication of the individual faecal microbiota of the 8 human adults (unique symbols) along with loading vectors for the 10 genera that explained most variation; (**B**) abundances (%) of the most abundant genera.

The eight test subjects exhibited distinct faecal microbiome profiles, which were preserved throughout the ex vivo incubations (Figure 2).

These interpersonal differences could be attributed to markers of the three enterotypes, previously introduced to describe variation in the human gut microbiota [44]. Most variation in the principal component analysis at the genus level (Figure 2A) was explained along PC1 (32.8%) and was differentiated between subjects with high *Prevotella* (left: subject 2 and especially the 1~*Prevotella* enterotype) and high *Ruminococcus* and/or *Coprococcus* levels (right: subjects 4/6/7). Further, PC2 explained 21.5% of the variation, mostly relating to elevated levels of *Faecalibacterium* in all subjects except donor 8, who had, in contrast, particularly elevated levels of *Agathobacter*. These last four genera belong to the Bacillota phylum (~Bacillota (formerly Firmicutes) enterotype). Subject 8 had particularly high levels of *Phocaeicola* and *Bacteroides* (~*Bacteroides* enterotype) (Figure 2B). Finally, *Bifidobacterium* levels were highest for subjects 5, 6, 8, and especially 7, further illustrating how the microbiomes employed in the present study captured interpersonal differences. This is essential to ensure that the findings of the study are representative at a human population level.

Another important aspect in obtaining representative findings is that the in vivo-derived microbiota are maintained during the full duration of the experiment. Despite strong increases in cell density (Figure S1D), both microbial diversity (Figure S1A–C) and composition (Figure S1E) were preserved throughout the 24 h incubation period. Maintenance of diversity was shown with indices for both species’ richness (Chao1 diversity index: Figure S1A) and evenness (reciprocal Simpson and Shannon diversity index: Figure S1B,C). These findings corroborated the biorelevance of the employed ex vivo incubation strategy and ensured that representative insights were obtained into how Bimuno GOS modulates the gut microbiome.

### 3.2. GOS Boosted SCFA Production at Only Mild Increases in Gas Production

Metabolite production in the ex vivo incubations was highly reproducible within subjects but showed notable interpersonal variation. First, the coefficient of variation between technical replicates (n = 3) of the NSC study arm at 24 h of incubation was as low as 1.3% for key fermentative parameters and confirmed the high technical reproducibility of the ex vivo incubations (within the test subject). In contrast, the coefficient of variation across the eight subjects was 8.8% reflecting interpersonal variation in metabolite production, in line with compositional differences at baseline.

**Figure 3:**
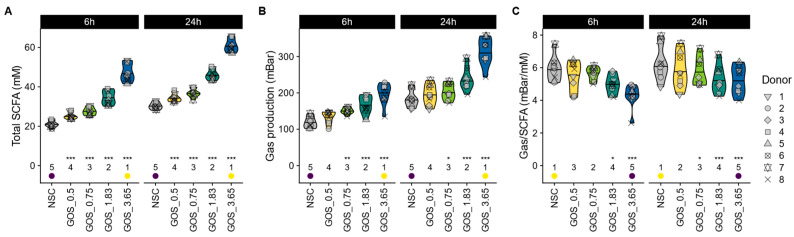
**GOS boosted SCFA production in a dose-related manner, which was accompanied by only mild increases in gas production.** The impact on (**A**) total SCFA (mM), (**B**) gas production (mbar), and (**C**) the ratio of gas being produced per mole of SCFA.

GOS was rapidly fermented by all donor microbiomes, leading to a dose-dependent increase in total SCFA levels as early as 6 h after incubation, with significant effects observed from 0.5 g (Figure 3A). GOS also increased gas production (Figure 3B) yet reduced the ratio of gas production per mole of SCFA being produced (Figure 3C). This indicates that the increase in gas production upon GOS supplementation was disproportionally low compared to the SCFA stimulation.

**Figure 4:**
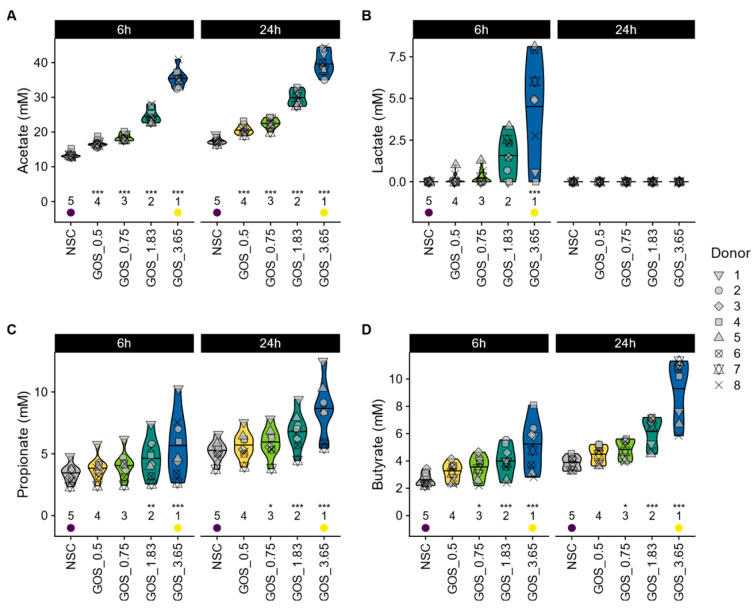
**GOS boosted production of the three main SCFAs and lactate in a dose-related manner.** The impact on (**A**) acetate (mM), (**B**) lactate (mM), (**C**) propionate (mM), and (**D**) butyrate (mM).

GOS differentially modulated SCFA profiles, with evidence of lactate cross-feeding to other SCFAs over time. When focusing on individual SCFAs, GOS were shown to significantly stimulate acetate from the lowest dose onwards (both at 6 h and 24 h) (Figure 4A), which was linked to statistically significant pH decreases for all test doses (Figure S2A). In contrast, lactate only increased significantly for the highest GOS dose at 6 h and was not detected at 24 h (Figure 4B), suggesting cross-feeding between 6 and 24 h in pathways that result in production of the SCFA propionate and/or butyrate (Figure 4C and Figure 4D, respectively). The latter increased significantly from a dose of 0.75 g onwards, both at 6 h and 24 h (except for propionate at 6 h) (Figure 4D). Finally, GOS also significantly reduced bCFA production for all test doses at 24 h (Figure S2B).

### 3.3. GOS Specifically Modulated Microbial Composition, Amongst Other Changes, Due to Strong Bifidogenic Effects

**Figure 5:**
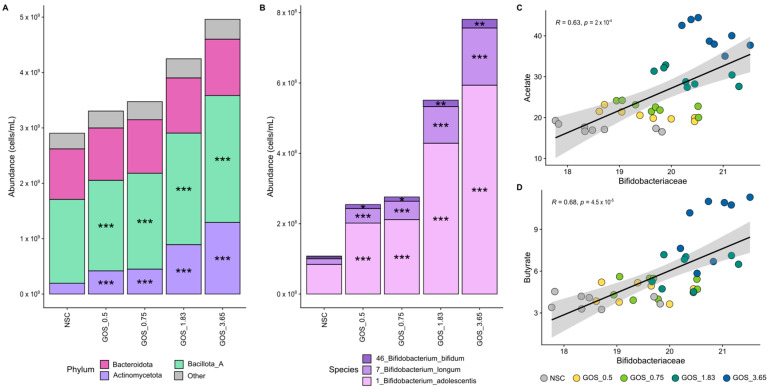
**GOS exerted significant bifidogenic effects from the lowest dose onwards, relating to enhanced acetate and butyrate production.** Average level (cells/mL) of (**A**) microbial phyla and (**B**) *Bifidobacteriaceae* (main family within Actinomycetota; deconvoluted in its main OTUs) (n = 8), along with correlations of absolute *Bifidobacteriaceae* levels with (**C**) acetate and (**D**) butyrate.

GOS supplementation dose-dependently shifted the microbiome composition, enriching Actinomycetota and Bacillota_A. Along with Bacteroidota, they represent the main phyla for the eight different subjects (Figure 5A). GOS significantly promoted Actinomycetota and Bacillota_A in a dose-dependent manner, from the lowest dose onwards. The subsequent result description will focus on the most abundant family (and related OTUs) of the three main phyla, with a complete analysis at the family and OTU levels being provided as Supplementary Information (Figures S3 and S4, respectively).

First, GOS strongly and dose-dependently enriched *Bifidobacteriaceae*, particularly *B. adolescentis*, correlating with increased acetate and butyrate production. The *Bifidobacteriaceae* family was the most abundant family within the Actinomycetota phylum, covering, on average, 90.5% of its abundance at 24 h. GOS stimulated *Bifidobacteriaceae* significantly from the lowest dose onwards, which related to dose-dependent and statistically significant increases of OTUs related to all three detected *Bifidobacterium* species, i.e., *B. bifidum, B. longum,* and particularly *B. adolescentis*. Strong and statistically significant correlations between *Bifidobacteriaceae* and both acetate (Figure 5C) and butyrate (Figure 5D) suggest the involvement of *Bifidobacteriaceae* in the production of these SCFAs upon GOS treatment.

**Figure 6:**
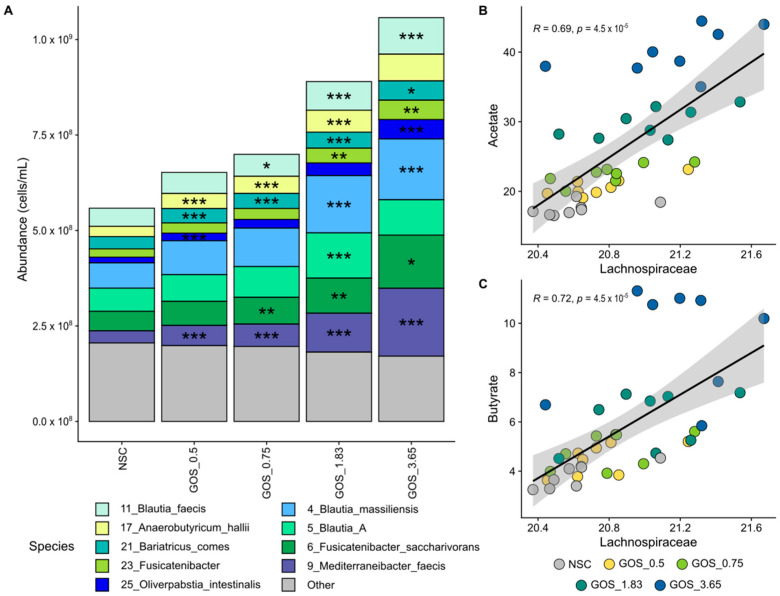
**GOS significantly stimulated *****Lachnospiraceae***** members from the lowest test dose onwards, relating to enhanced acetate and butyrate production.** Average level (cells/mL) of (**A**) *Lachnospiraceae* (main family within Bacillota_A; deconvoluted in its main OTUs) (n = 8), along with correlations of absolute *Lachnospiraceae* levels with (**B**) acetate and (**C**) butyrate.

GOS dose-dependently enriched *Lachnospiraceae*, with multiple contributing species, correlating with enhanced acetate and butyrate production. *Lachnospiraceae* was the most abundant Bacillota_A family, accounting, on average, for 68.0% of its abundance at 24 h. GOS stimulated *Lachnospiraceae* significantly from the lowest test dose on, which related to dose-dependent and statistically significant increases of OTUs related to *Anaerobutyricum hallii*, *Bariatricus comes*, *Blautia* species (*B. massiliensis*, *Blautia_A*, *B. faecis)*, *Oliverpabstia intestinalis*, *Mediterraneibacter faeciss* and *Fusicatenibacter* species (e.g., *F. saccharivorans*) (Figure 6A). Strong and statistically significant correlations between *Lachnospiraceae* and both acetate (Figure 6B) and butyrate (Figure 6C) suggest the involvement of *Lachnospiraceae* in the production of these SCFAs upon GOS treatment.

**Figure 7:**
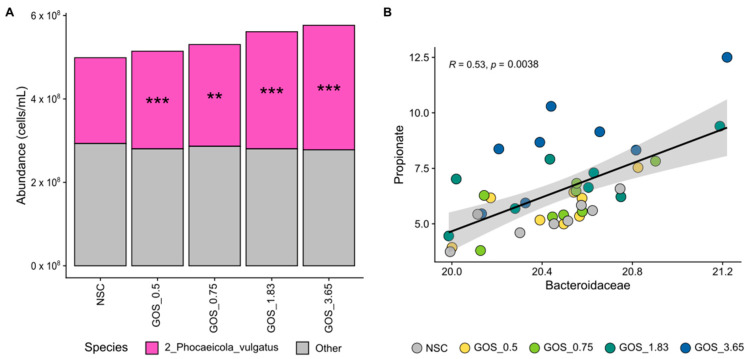
**GOS significantly stimulated an OTU related to *****Phocaeicola vulgatus*****, an abundant *****Bacteroidaceae***** member, relating to enhanced propionate production.** Average level (cells/mL) of (**A**) *Bacteroidaceae* (main family within Bacteroidota; deconvoluted in its main OTUs) (n = 8), along with (**B**) correlations between absolute *Bacteroidaceae* levels and propionate.

Finally, GOS selectively enriched *Bacteroidaceae*, particularly *Phocaeicola vulgatus*, which correlated with increased propionate production. *Bacteroidaceae* was the most abundant Bacteroidota family, covering, on average, 83.4% of its abundance at 24 h. GOS significantly stimulated an OTU related to *Phocaeicola vulgatus*, an abundant member of this family, from the lowest test dose onwards (Figure 7A). The abundance of this OTU correlated with propionate levels (Figure 7B), suggesting the involvement of *P. vulgatus* in propionate production upon GOS treatment.

## 4. Discussion

The current SIFR^®^ study was conducted under optimal conditions, showing high technical reproducibility and preserving the microbial diversity and composition of healthy adult microbiomes throughout the experiment. This enabled representative insights into the potential clinical effectiveness of ultra-low, capsule-compatible doses of Bimuno^®^ GOS. The GOS preparation was rapidly fermented with strong effects on key fermentative parameters within 6 h. Observations between 0–6 h are representative for findings of the ascending colon [45], suggesting that the GOS preparation exerts instant effects in the proximal colon.

We demonstrated that from a dose of 0.5 g onwards, the GOS preparation significantly promoted the abundance of three *Bifidobacterium* species (*B. adolescentis*, *B. longum,* and *B. bifidum*) as well as the production of acetate, the main end-metabolite of carbohydrate fermentation by bifidobacteria [46]. GOS have indeed demonstrated bifidogenic effects across diverse populations, including infants [47,48], healthy adults [13,14,37], the elderly [49], and in individuals with specific deficiencies or disorders [50,51]. Besides exerting benefits via the production of acetate [52], *Bifidobacterium* species exert health benefits via other metabolites including tryptophan metabolites like aromatic lactic acids [53]. Kumar et al. (2020) [54] reported that low *Bifidobacteriaceae* levels in adults are associated with allergies and other diseases, often resulting from early-life events (e.g., C-section) or factors such as diet, lifestyle, or antibiotic use. This renders *Bifidobacteriaceae* interesting targets when aiming to maintain or restore health via microbiome modulation strategies such as GOS supplementation.

Adding to the current knowledge, GOS not only stimulated *Bifidobacteriaceae* and acetate but also promoted a consortium of *Lachnospiraceae* species. Three species were already significantly promoted from a dose of 0.5 g onwards, i.e., *Anaerobutyricum hallii* (formerly known as *Eubacterium hallii*, a species known to convert *Bifidobacterium*-derived metabolites (lactate and acetate) into propionate [55] and especially butyrate [56]), *Bariatricus comes* (formerly known as *Coprococcus comes*, a known butyrate producer [52]), and *Mediterraneibacter faecis* (formerly known as *Ruminococcus faecis* [57]). Other *Lachnospiraceae* members that significantly increased from a dose of 1.83 g onwards included several *Blautia* species, the recently isolated *Oliverpabstia intestinalis* [58], and *Fusicatenibacter saccharivorans* [59]. The latter was previously identified as a discriminator between healthy patients and those with ulcerative colitis, potentially playing a pivotal role due to its IL-10-mediated anti-inflammatory effects [60]. Because *Lachnospiraceae* strongly correlated with butyrate levels during GOS treatment, these taxa likely contributed to butyrate production, an SCFA that fuels epithelial cells and helps protect against cancer and colitis [52]. Overall, GOS thus also positively affect the microbiome beyond just the bifidogenic effects, benefiting a variety of butyrate producers even at low doses.

Further, GOS also promoted propionate production by *Phocaeicola vulgatus*. Propionate exerts benefits by promoting satiety, lowering blood cholesterol, and improving insulin sensitivity [52]. However, *P. vulgatus* gained attention for its ability to promote the secretion of GLP-1 via its metabolite pantothenate, leading to a lower sugar preference, thus rendering *P.-vulgatus*-derived pantothenate as a target for diabetes [61]. This further adds to the evidence that GOS positively affect the microbiome beyond just bifidogenic effects.

The potent microbiome modulation by GOS at ultra-low doses, uncovered in the present study, aligns with effective doses reported in clinical studies. Indeed, while studied GOS doses range from 1.3–2.5 g/day [13,37,62] up to 10–15 g/day [15,20,37], studies with doses within the lower dose range have reported significant effects. For example, Lee et al. (2024) demonstrated improved bowel habits and increased levels of *Bifidobacterium* species following the daily intake of 2 g of GOS over 4 weeks [62]. This suggests that the minimally effective dose of GOS (i.e., the lowest intake yielding significant beneficial effects) may be lower than the minimally tested doses in these studies and could be in the range of 0.5 g, a dose that significantly modulated the microbiome ex vivo in the present study.

A side finding of the study was that the strong increase in SCFA production with GOS was only accompanied by low gas production, suggesting a high tolerability of GOS for human consumption. The ratio of gas production per mole of SCFAs being produced decreased significantly from a dose of 0.75 g onwards. This matches findings from a clinical study where 2.75 g/day of Bimuno^®^ GOS reduced bloating, flatulence, and abdominal pain in adults within one week [17]. Mechanistically, this could relate to the potent bifidogenic effects of GOS. As reviewed by Rivière et al. (2016) [52], *Bifidobacterium* species employ the fructose-6-phosphate phosphoketolase pathway (or bifid shunt) that does not result in gas production, causing acid production from carbohydrates in the absence of gas formation [63]. In contrast, butyrate producers do produce gases (H_2_ and/or CO_2_) [52], explaining the mild increase in gas production for GOS. Yet, the gas production was likely attenuated by the bifidogenic effect given the findings of Moens et al. (2017), who demonstrated that the production of acetate by a *Bifidobacterium* species (*B. longum* LMG 11047) lowers gas (H_2_) production during butyrate formation by, for instance, *Anaerobutyricum hallii* [56]. This low gas production with GOS also aligns with benefits that have been observed upon GOS intake in the context of managing lactose intolerance [64]. Mechanistically, the β-galactosidase activity employed by *Bifidobacterium* species to ferment GOS can also be used to ferment lactose. Hence, bifidogenic effects by GOS could explain why the microbiome is able to ferment lactose with lower side effects.

Many of the findings of the present study were enabled using the recently developed ex vivo SIFR^®^ technology. Indeed, this study revealed broader microbiome effects of Bimuno^®^ GOS than previous in vitro studies, which only detected acetate and *Bifidobacteriaceae* changes, despite using higher doses (3.5–5.5 g). As has been discussed previously [38], the SIFR^®^ technology is highly reproducible and closely maintains the original in vivo microbiota, thus also preserving strict anaerobes like hard-to-culture butyrate producers. This contrasts with in vitro gut models where a large amount of diversity gets lost during incubation, both in short-term [33] and long-term gut models [65]. The unique features of the SIFR^®^ technology explain how, within days of the experiment, findings are obtained that are predictive for those of clinical studies where the same substrate is consumed repeatedly over weeks.

Finally, it should be noted that ex vivo microbiome studies also have limitations, such as the absence of host factors, which means that clinical trials remain important to prove the clinical efficacy of low doses of GOS.

## 5. Conclusions

Overall, the present study highlights the promising clinical potential of ultra-low, capsule-compatible doses of Bimuno^®^ GOS. Incorporating GOS into capsules or tablets not only enhances convenience and flexibility in dietary supplement design but also offers practical advantages for consumers. Notably, larger doses could be more feasibly administered throughout the day via capsules or tablets rather than as single daily sachets. For instance, 5.5 g (48% GOS) has been proven to be effective for the management of traveller’s diarrhoea [32], yet it could be more conveniently taken as capsules or tablets throughout the day instead of as a single daily sachet in order to create more convenience while traveling. On the other hand, spreading GOS ingestion throughout the day could also cause a more sustained treatment effect by more regularly feeding the microbiome rather than in pulses once a day. Fractionated dosing may be especially beneficial for sensitive populations, such as individuals with IBS, who often have a low tolerance for fermentable prebiotics. Administering smaller doses multiple times a day could allow patients with IBS to increase their total GOS intake beyond the standard daily dose without triggering adverse effects. Collectively, these findings open avenues for the development and evaluation of alternative dosing strategies that optimize the clinical effectiveness of prebiotics.

## Data Availability

The datasets generated during and/or analysed during the current study are available from the corresponding author upon reasonable request.

## Supplementary Materials

The following supporting information can be downloaded at: https://www.mdpi.com/article/10.3390/metabo15080530/s1, Figure S1: The diversity and composition of the gut microbiome was maintained along the full duration of the ex vivo SIFR^®^ experiment; Figure S2: GOS significantly reduced pH and inhibited bCFA production in a dose-related manner; Figure S3: GOS mainly stimulated *Bifidobacteriaceae*, *Lachnospiraceae,* and *Bacteroidaceae*, from the lowest test dose onwards; Figure S4: GOS mainly stimulated OTUs belonging to the *Bifidobacteriaceae*, *Lachnospiraceae,* and *Bacteroidaceae* families, from the lowest test dose onwards.

## Abbreviations

The following abbreviations are used in this manuscript:

bCFA	Branched-chain fatty acid
BSS	Bristol stool score
GOS	Galacto-oligosaccharide
IBS	Irritable bowel syndrome
NSC	No substrate control
SCFA	Short-chain fatty acid
SIFR^®^	Systemic intestinal fermentation research
