# Supplementary material for: Galacto-Oligosaccharides Exert Bifidogenic Effects at Capsule-Compatible Ultra-Low Doses
Source: Metabolites. 2025 Aug 5;15(8):530. doi: 10.3390/metabo15080530 (PMC12388037; doi:10.3390/metabo15080530)
Supplement: Supplementary file 1 [file metabolites-15-00530-s001.zip › metabolites-3752503-supplementary.pdf]

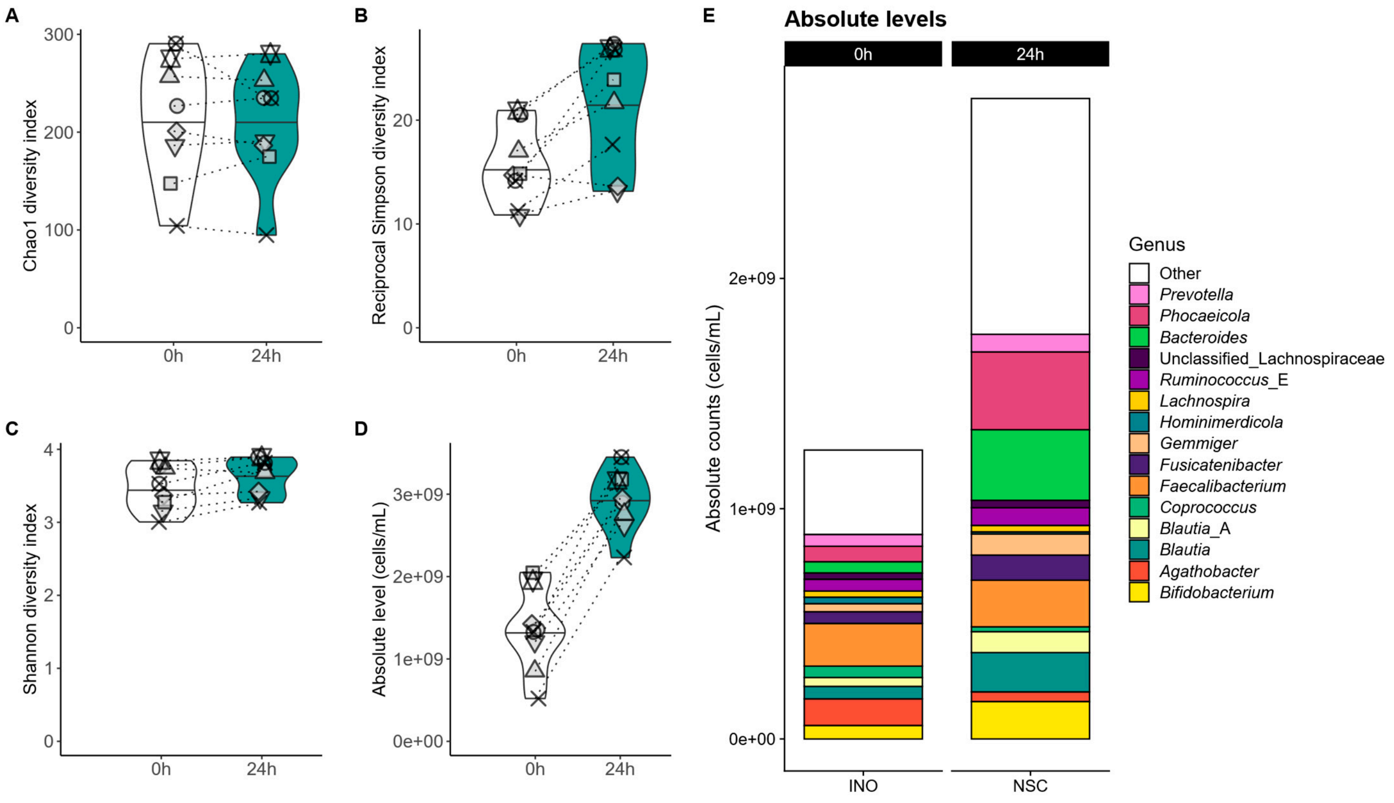

Figure S1. The diversity and composition of the gut microbiome was maintained along the full duration of the *ex vivo* SIFR® experiment. (A/B/C) Microbial diversity in terms of species' richness (Chao1 diversity index) and evenness (reciprocal Simpson and Shannon diversity index), (D) bacterial cell density (cells/mL) and (E) average microbial composition at genus level (cells/mL) of the *in vivo*-derived microbiota at baseline (INO) and upon 24h of incubation in the SIFR® technology in absence of a treatment (NSC) (n = 8).

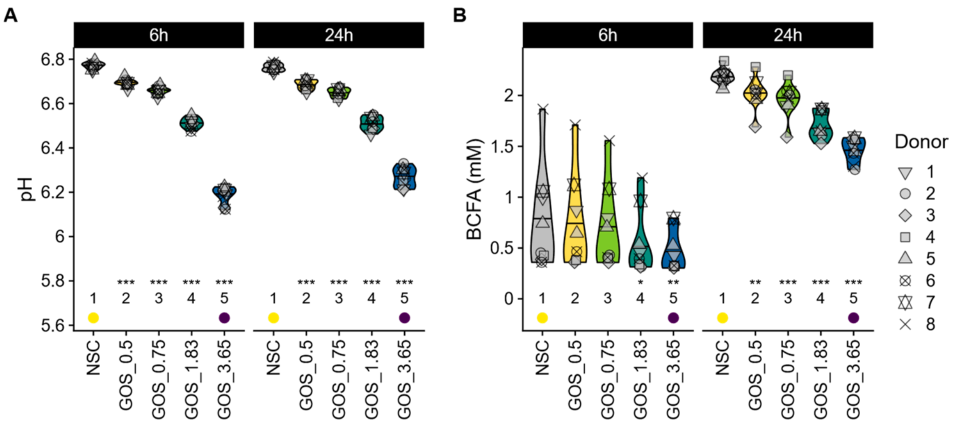

Figure S2. GOS significantly reduced pH and inhibited bCFA production in a dose-related manner. The impact on (A) pH and (B) bCFA (mM). Statistical differences between specific doses of GOS and the NSC are indicated with * ($0.01 < p_{adjusted} < 0.05$), ** ($0.001 < p_{adjusted} < 0.01$) or *** ($p_{adjusted} < 0.001$). The ranks of average values per study arm are indicated below the statistical indicator, with the lowest and highest value being highlighted in purple and yellow, respectively.

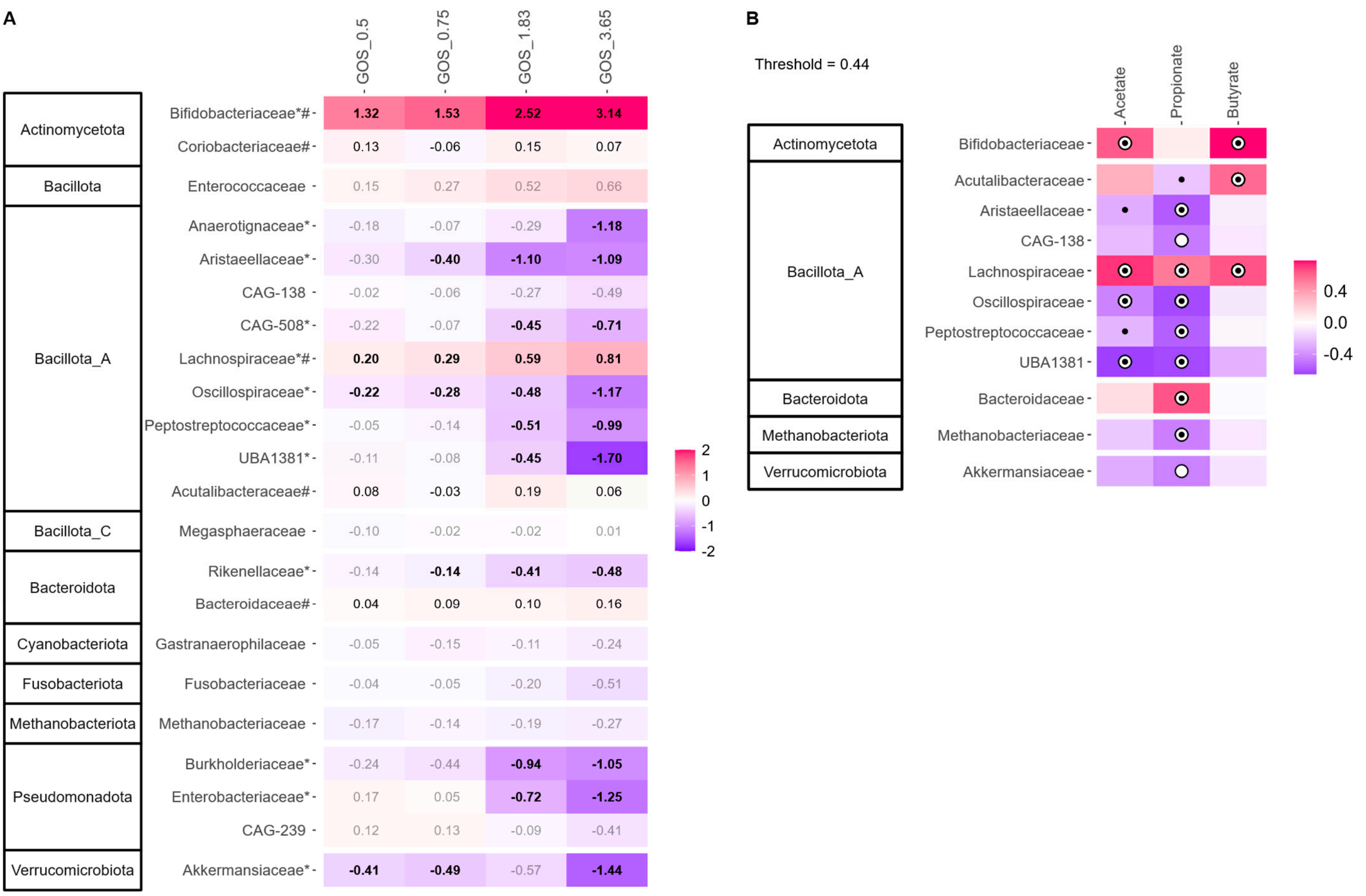

Figure S3. GOS mainly stimulated *Bifidobacteriaceae, Lachnospiraceae* and *Bacteroidaceae,* from the lowest test dose onwards. While *Bifidobacteriaceae* and *Lachnospiraceae* particularly correlated with increased acetate and butyrate production, *Bacteroidaceae* correlated with propionate production. (A) The impact on families that were significantly ($p_{adjusted} < 0.05$, indicated by *) affected by any of the treatments, expressed as log_2 transformation of abundance treatment/abundance NSC ratios, averaged across all 8 test subjects, at 24h after initiation of the colonic incubations. Values were indicated in bold were statistically significant increase/decrease occurred. Top 5 families with the largest variations among the treatments were also included in the heat map and indicated with #. (B) Regularised Canonical Correlation Analysis (rCCA) to highlight correlations between fundamental fermentative parameters and microbial composition (threshold: R-value of correlation > 0.44). The white circles indicate values larger than the threshold and black dots indicate statistical significance ($p < 0.05$) in the individual correlations between the SCFA and the families based on Spearman's rank correlation coefficient.

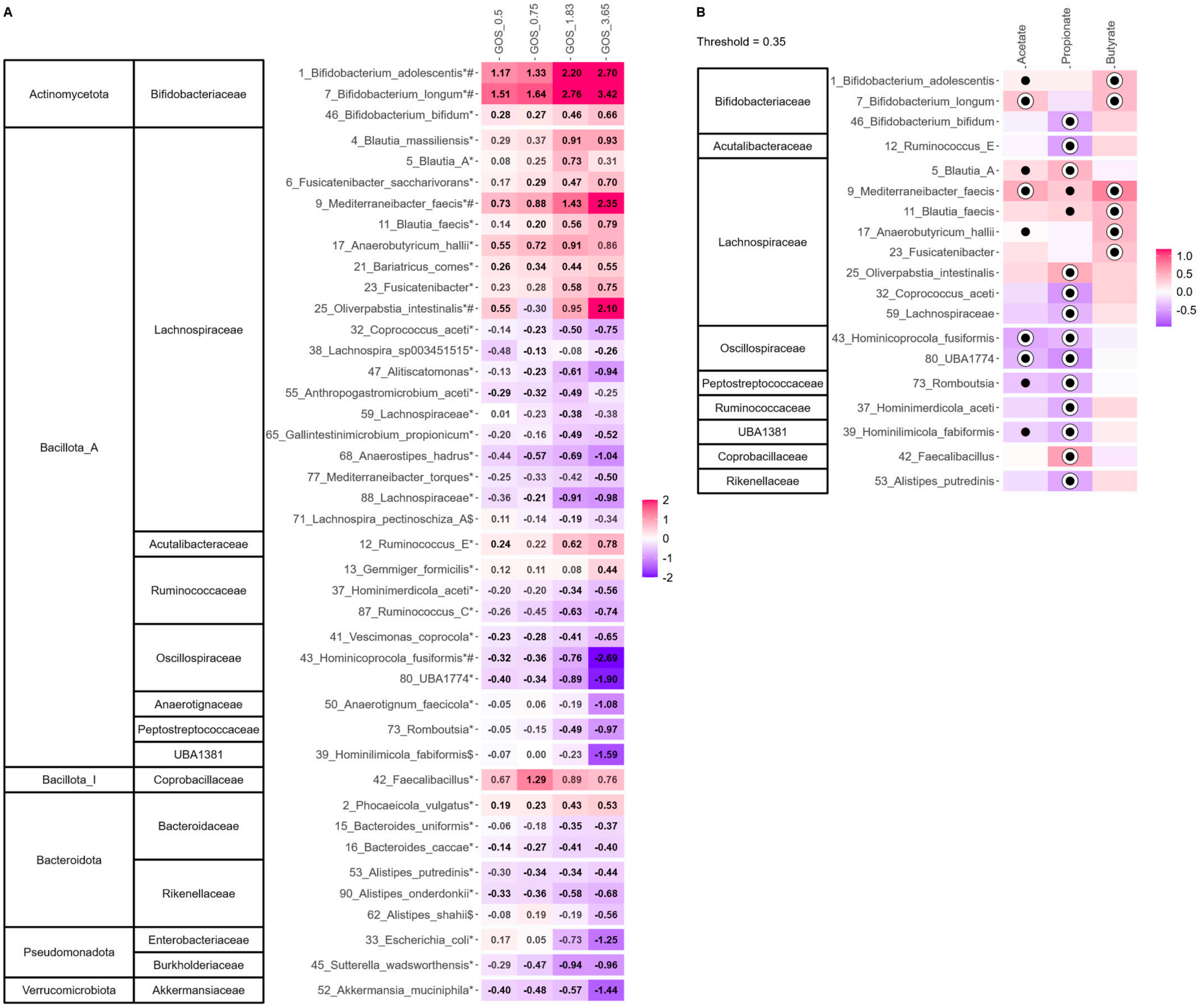

Figure S4. GOS mainly stimulated OTUs belonging to the *Bifidobacteriaceae, Lachnospiraceae* and *Bacteroidaceae* families, from the lowest test dose onwards. Several *Bifidobacterium* and various *Lachnospiraceae* species particularly correlated with acetate and butyrate production. (A) The impact on OTUs that were significantly ($p_{adjusted} < 0.05$, indicated by *) affected by any of the treatments, expressed as log_2 transformation of abundance treatment/abundance NSC ratios, averaged across all 8 test subjects, at 24h after initiation of the colonic incubations. Values were indicated in bold were statistically significant increase/decrease occurred. Top 5 OTUs with the largest variations among the treatments were also included in the heat map and indicated with #. (B) Regularised Canonical Correlation Analysis (rCCA) to highlight correlations between fundamental fermentative parameters and microbial composition (threshold: R-value of correlation > 0.35). The white circles indicate values larger than the threshold and black dots indicate statistical significance ($p < 0.05$) in the individual correlations between the SCFA and the OTUs based on Spearman's rank correlation coefficient.
